# Administration of Panobinostat Is Associated with Increased IL-17A mRNA in the Intestinal Epithelium of HIV-1 Patients

**DOI:** 10.1155/2015/120605

**Published:** 2015-12-01

**Authors:** Ane Bjerg Christensen, Anders Dige, Johan Vad-Nielsen, Christel R. Brinkmann, Mia Bendix, Lars Østergaard, Martin Tolstrup, Ole S. Søgaard, Thomas A. Rasmussen, Jens Randel Nyengaard, Jørgen Agnholt, Paul W. Denton

**Affiliations:** ^1^Department of Infectious Diseases, Aarhus University Hospital, 8200 Skejby, Denmark; ^2^Department of Hepatology and Gastroenterology, Aarhus University Hospital, 8000 Aarhus, Denmark; ^3^Department of Clinical Medicine, Aarhus University, 8000 Aarhus, Denmark; ^4^Stereology and Electron Microscopy Laboratory, Centre for Stochastic Geometry and Advanced Bioimaging, Aarhus University Hospital, 8000 Aarhus, Denmark; ^5^Aarhus Institute for Advanced Studies, Aarhus University, 8000 Aarhus, Denmark

## Abstract

Intestinal CD4^+^ T cell depletion is rapid and profound during early HIV-1 infection. This leads to a compromised mucosal barrier that prompts chronic systemic inflammation. The preferential loss of intestinal T helper 17 (Th17) cells in HIV-1 disease is a driver of the damage within the mucosal barrier and of disease progression. Thus, understanding the effects of new therapeutic strategies in the intestines has high priority. Histone deacetylase (HDAC) inhibitors (e.g., panobinostat) are actively under investigation as potential latency reversing agents in HIV eradication studies. These drugs have broad effects that go beyond reactivating virus, including modulation of immune pathways. We examined colonic biopsies from ART suppressed HIV-1 infected individuals (clinicaltrials.gov: NCT01680094) for the effects of panobinostat on intestinal T cell activation and on inflammatory cytokine production. We compared biopsy samples that were collected before and during oral panobinostat treatment and observed that panobinostat had a clear biological impact in this anatomical compartment. Specifically, we observed a decrease in CD69^+^ intestinal lamina propria T cell frequency and increased IL-17A mRNA expression in the intestinal epithelium. These results suggest that panobinostat therapy may influence the restoration of mucosal barrier function in these patients.

## 1. Introduction

Intestines play a major role in maintaining our health. Beyond nutrient and liquid absorption, the intestines serve as a barrier between us, our intestinal microbiome, and foreign organisms/toxins. It is therefore expected that significant morbidity and mortality result from intestinal disorders like those associated with abnormal immune function (e.g., inflammatory bowel disease) and infectious diseases (e.g., various foodborne illnesses) [1, 2]. HIV-1 is an infectious disease that has a major negative impact on the intestines. Viral infiltration and replication in early HIV-1 infection result in rapid and profound intestinal CD4^+^ T cell depletion [3–10]. This leads to a compromised mucosal barrier and then to chronic systemic inflammation [11]. The preferential loss of intestinal T helper 17 (Th17) cells in HIV disease is a driver of the damage within the mucosal barrier and this damage is not reversed during antiretroviral therapy (ART) [12]. A key effector molecule produced by Th17 cells (as well as *γδ* T cells and a subset of innate lymphoid cells) is IL-17A. IL-17A is a multifunctional cytokine with proinflammatory properties (e.g., neutrophil recruitment) along with a role in orchestrating mucosal barrier functions [13–15]. Given the pathogenesis of HIV-1 in the intestines, understanding the effects of new therapeutic strategies within this organ is a priority.

Recently, we conducted a single-arm, phase I/II clinical trial designed to evaluate the therapeutic effect of the HDAC inhibitor panobinostat on HIV-1 persistence despite successful ART [16]. Panobinostat is a potent hydroxamic acid HDAC inhibitor with inhibitory effects in the low nanomolar range against class I HDAC. It was approved in 2015 by the US Food and Drug Administration for the treatment of multiple myeloma [17]. HDAC inhibitors, like panobinostat, are actively under investigation as potential latency reversing agents because HIV-1 proviruses that are integrated into deacetylated, condensed chromatin lead to virus recrudescence when ART is interrupted. Accordingly, HDAC inhibitors have been extensively studied* in vitro* [18–20] and* in vivo* [16, 21–23] for their latency reversing potential and the results have been very promising. Furthermore, many HDAC inhibitors have robust anti-inflammatory properties [24]. These effects were observed in our trial cohort where panobinostat treatment was associated with reduced levels of peripheral blood (PB) inflammatory biomarkers (e.g., high-sensitivity C-reactive protein, interleukin-6, matrix metalloproteinase 9, E-selectin, and soluble CD40 ligand) as well as reduced expression of genes related to inflammation [25]. Because of the importance of understanding the varied intestinal effects of HDAC inhibitors as HIV-1 therapeutics, the study design included the collection of intestinal biopsies from consenting participants. These paired biopsies are the source material for this study to quantitate the biological impact of panobinostat in the intestines of individuals during suppressive ART.

## 2. Methods

### 2.1. Study Design and Participants

**Figure 1:**
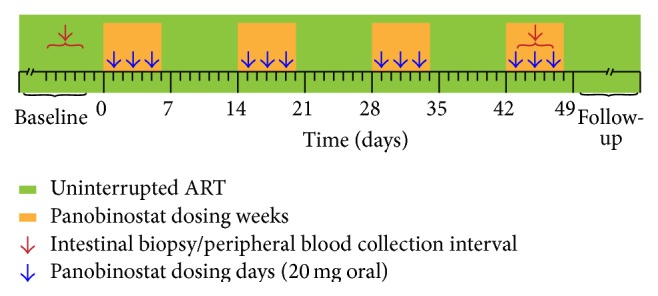
Schematic representation of the trial design. The timing of panobinostat dosing and the collection of samples utilized in these analyses are illustrated.

Between September 2012 and February 2014 we conducted an investigator-initiated, single-arm, phase I/II clinical trial as previously described (clinicaltrials.gov ID number NCT01680094) [16]. In accordance with the principles of the Helsinki Declaration, the Regional Ethics Committee for Region Midtjylland and the Danish Data Protection Agency approved the study design prior to patient enrollment and each patient provided written informed consent before any study procedures. Fifteen HIV-1 infected adults were enrolled in the study. These individuals exhibited virological suppression (<50 copies per mL, at least two measurements per year) for at least 2 years and CD4^+^ T cell counts above 500 cells per *μ*L. Each patient received oral panobinostat 20 mg three times per week every other week for eight weeks while maintaining ART (Figure 1). Study exclusion criteria included coinfection with hepatitis B or C viruses, clinically significant cardiac disease (including QTc prolongation), and current use of a protease inhibitor (because of potential drug interactions). Of the 15 patients, 9 individuals (all infected with Clade B virus) consented to participate in an endoscopic substudy with collection of mucosal biopsies from the sigmoid colon in the week prior to panobinostat dosing and during the fourth dosing week (Figure 1). The biopsies from the fourth week of panobinostat treatment period were collected between 22 and 24 hours after the most recent oral panobinostat dose.

### 2.2. Sigmoid Biopsies and Isolation of Lamina Propria Mononuclear Cells (LPMCs)

Sigmoidoscopy was performed by the same experienced endoscopists at baseline and during the fourth week of panobinostat treatment. An Olympus Exera II CLV-180 with an Olympus GIF-H180 scope (Olympus, Tokyo, Japan) was used to perform the procedures. The sigmoidoscopies were done without prior bowel cleaning. During all endoscopies mucosal tissue samples were taken from a standardized location in the sigmoid colon (approximately 35 cm from the anal verge). Endoscopic biopsies were collected for multiple analyses from macroscopically normal intestinal mucosa. Two biopsies were randomly selected to be fixed in 4% PFA (6–12 hours at room temperature) and embedded in paraffin blocks for gross histopathology. The remaining biopsies were collected in ice-cold Dulbecco's phosphate buffered saline (PBS) and immediately placed on ice. These biopsies were then processed to obtain lamina propria mononuclear cells (LPMCs). First, epithelial cells were removed by three 15 min incubations at 37°C in CMF HBSS-EDTA (calcium-magnesium free Hank's buffered salt solution supplemented with 2% human AB serum, 1.5 mM HEPES (Gibco Life Technologies, Auckland, New Zealand), and 2 mM EDTA (Thermo Fisher Scientific/Ambion, Waltham, Massachusetts)). After each incubation period, the epithelial cell-containing CMF HBSS-EDTA was removed and discarded. Following the third CMF HBSS-EDTA incubation the tissue samples were washed in RPMI 1640 supplemented with 10% AB serum and 1.5 mM HEPES. LPMCs were then isolated via a 90 min incubation at 37°C in 5 mL digestion media (RPMI 1640 supplemented with 10% human AB serum, 1.5 mM HEPES, 0.1 mg of collagenase D (Sigma-Aldrich, St. Louis, Missouri), and 50 U/mL DNase I (Sigma-Aldrich)). Following digestion, LPMC-containing supernatants were collected via filtration through a 70 *μ*m nylon mesh (BD Biosciences, San Jose, California). Viable cells were counted and then aliquoted for quantitative PCR and flow cytometry analyses.

### 2.3. Quantification of Viral DNA

For four patients (identified as ▲, ●, ■, and *◆* in figures), LPMCs were used to isolate CD4^+^ T cells using a CD4^+^ T cell isolation kit (Miltenyi Biotec, #130-096-533) and magnetic-activated cell sorting (MACS) columns (purity > 95%). The goal was to gain more specific insights into these potential viral reservoir cells. However, the total cell yields from these four isolations were low and therefore CD4^+^ T cell enrichment was not performed with biopsies from the remaining five patients (identified as ∆, *⚪*, □, ×, and ★ in figures). To compare anatomical compartments, PB CD4^+^ T cells collected on the nearest date to the time of biopsy were analyzed for viral DNA load by the same method used for the LPMCs. Isolated LPMCs, LP CD4^+^ T cells, and PB CD4^+^ T cells to be used for nucleic acid quantification were lysed in RLT Plus Buffer immediately following isolation and the lysates were stored at −80°C until DNA was extracted (Allprep isolation kit, Qiagen #80204). Just prior to extraction, the lysates were subjected to shredding (Qiashredder, Qiagen #79656) and then nucleic acids were extracted according to manufacturer's instructions with each nucleic acid sample being eluted with three repeated applications of the same 50 *μ*L elution buffer aliquot to their respective columns.

HIV-1 DNA was quantified in samples obtained from both the intestinal biopsies and PB cells at baseline and on panobinostat essentially as described [26, 27]. Briefly, extracted DNA was used directly for HIV-1 DNA quantifications using the QX100 Droplet Digital PCR system (BioRad) (~100 ng total DNA per PCR replicate) to determine the absolute levels of total HIV-1 DNA per 10^6^ LPMCs, LP CD4^+^ T cells, or PB CD4^+^ T cells. The PCR reaction mixture was loaded into the BioRad QX100 emulsification device fractionating each sample into ~20,000 nanoliter-sized droplets according to the manufacturer's instructions. After cycling, droplet data were collected using QX100 droplet reader (BioRad) and then analyzed with the QuantaSoft analysis software (BioRad). No panobinostat-associated cohort-wide changes in HIV-1 DNA levels were observed in intestinal cells (*p* = 0.91) (similar to [23]) or in time-matched PB CD4^+^ T cells (*p* = 0.57) (consistent with our previous report [16]).

### 2.4. Cell Surface and Intracellular Cytokine Flow Cytometry Analyses

LPMCs surface expression of the T cell activation markers was measured essentially as described [28]. Briefly, within one hour of the isolation, cells were incubated with heat-inactivated mouse serum (Invitrogen, cat. number 10410) for 10 min and then stained with CD3 PerCP (BD Biosciences, cat. number 345766), CD4 PE (BD Pharmingen, cat. number 555347), CD8 PE-Cy7 (BD Pharmingen, cat. number 557746), CD69 APC (Biolegend, cat. number 310910), and HLA-DR APC-Cy7 (Biolegend, cat. number 307618). Following staining, the cells were fixed in 250 *μ*L PBS containing 1% formaldehyde.

Intracellular cytokine detection in LPMCs was performed essentially as described [28]. Briefly, freshly isolated cells were incubated unstimulated overnight at 37°C in flat-bottomed wells (Nunc, Denmark, cat. number 140675) at a concentration of 2 × 10^6^ LPMCs/mL in 2 mL of culture medium (RPMI 1640 with 10% pooled heat-inactivated human AB serum, 100 U/mL penicillin, and 100 *μ*g/mL streptomycin). Following this incubation, the cells were stimulated for 4 hours at 37°C with 1 *μ*g/mL ionomycin (Sigma-Aldrich, Denmark, cat. number I0634) and 50 *μ*g/mL phorbol-12-myristate-13-acetate (PMA) (Sigma-Aldrich, Denmark, cat. number P1585) in the presence of 10 *μ*g/mL brefeldin A (Sigma-Aldrich, Denmark, cat. number B7651). Next, cells were incubated with antibodies to detect surface expression of CD3 FITC (BD Biosciences, cat. number 345764) and CD8 PE-Cy7 (BD Pharmingen, cat. number 557746). Following surface staining, the cells were fixed with 1.5 mL BD FACS Lysing Solution (BD Biosciences, cat. number 349202) and then the cells were permeabilized with 0.5 mL FACS Permeabilizing Solution 2 (BD Biosciences, cat. number 340973). Blocking was performed with heat-inactivated mouse serum (Invitrogen, cat. number 10410) prior to staining with IFN-*γ* PE (eBioscience, cat. number 12-7319) and anti-IL-17A APC (eBioscience, cat. number 17-7179-42). Following intracellular staining, the cells were fixed in 250 *μ*L PBS containing 1% formaldehyde.

For all samples, data were collected using a FACSCanto analyzer (BD Biosciences) within 24 hours of staining. A total of 10^5^ events in the forward-side scatter lymphocyte gate were recorded. Single cells (according to forward-scatter-height and forward-scatter-area) were gated for CD3 expression. CD3^+^ cells were next gated for CD4^+^, CD8^+^, or CD8^neg^, as indicated in the figures. The cell populations were then assessed for expression of cellular activation markers and intracellular cytokines. Gates for CD69-, HLA-DR-, IFN-*γ*-, and IL-17A-positive events were based upon isotype or fluorescence-minus-one controls. Data from each patient from both time points were batch analyzed using FlowJo v.10 (Treestar).

### 2.5. *In Situ* Hybridization (ISH)

*In situ* hybridization for IFN-*γ*, IL-1*β*, IL-8, and IL-17A mRNA as well as for HIV-1 RNA was performed in formalin-fixed paraffin-embedded (FFPE) tissues using the RNAScope 2.0 RED assay (cat. number 310036) according to the manufacturer's instructions (Advanced Cell Diagnostics, Inc., Hayward, CA, USA). First, 4 *μ*m sections were cut from FFPE blocks and mounted on Superfrost Plus microscope slides. Consecutive sections were mounted and a minimum separation distance of 12 *μ*m was ensured for the sections hybridized with a given probe (2 sections per probe). Slides were placed at 60°C for 1 hr in a dry oven. Mounted sections were deparaffinized in xylene, dehydrated in 100% ethanol, and then treated serially with ACD Pretreatment 1 (endogenous hydrogen peroxidase block) for 10 minutes at room temperature; ACD Pretreatment 2 (boiling in citrate buffer) for 8–12 minutes at 98–100°C; and ACD Pretreatment 3 (protease digestion) for 12–30 minutes at 40°C. Note that Pretreatment 2 and 3 conditions for each paraffin block were optimized to maximize the signal-to-noise ratio using positive (Hs-PPIB cat. number 313901) and negative (DapB cat. number 310043) control RNA probes. Washes with deionized water were performed after each pretreatment step. Next, hybridization between the target RNA and the selected ACD probe set (Hs-IL-17A cat. number 310931; Hs-IFN-*γ* cat. number 310501; Hs-IL-8 cat. number 310381; Hs-IL1*β* cat. number 310361; V-HIV1-CladeB cat. number 416111) took place at 40°C during a 2-hour incubation in a HybEZ Oven (Advanced Cell Diagnostics, Hayward, CA). Two 2 min washes with ACD wash buffer were followed by signal amplification via the serial application of Amplifications 1–6 with two 2 min wash buffer washes after each step. Chromogenic detection of the target RNA was performed using Fast Red (10 min) followed by counterstaining with hematoxylin and mounting with Ecomount.

### 2.6. Quantification of ISH Signals

All of the sections were blinded before quantification and all counts were performed by the same individual via visual inspection with compound light microscopy with a robotic stage that randomly selected areas for quantification (Visiopharm NewCast, version 5.0.3.1247). In a minimum of 35 systematic random selected, nonoverlaying 25.600 *μ*m^2^ (160 *μ*m × 160 *μ*m) counting frames the number of positive cell profiles was counted. Test points were used to determine the area of examined tissue and the nature of the tissues within the counting frame (e.g., epithelial region, lamina propria) was recorded to allow for stratification of the data by anatomical region. In accordance with a recommendation from ACD, a cell profile was defined as RNA positive if it had at least one red spot visible at 20x magnification. Ratios were calculated for each of the probes and the background was subtracted. ISH data are expressed as positive cell profiles per mm^2^. Validation of the ISH quantification strategy was performed according to standard methods. Briefly, all sections were counted prior to unblinding. After all sections were counted, 25 sections that covered all probe sets were assigned for recount prior to unblinding of the individual performing the quantification. These two count values for the same section (C1 and C2) were first plotted in a C1/C2 plot and a linear regression was performed (*r* ^2^ = 0.36). Next, the C1 and C2 values were compared using an unpaired *t*-test (*p* = 0.31). Finally, a Bland-Altman plot was generated using the average of the two counts versus the difference between the two counts (bias = 19.1; SD = 59.5). Together these values demonstrate that consistency in performing the quantification was maintained throughout the entire counting process. Note that the area measurements reported will not be the same as the true parameters present* in vivo* given that paraffin embedding and preparation caused an unavoidable, 30–50% shrinkage of tissue [29, 30]. One goal in this project was to quantify the number of cell profiles exhibiting productive HIV-1 infection via ISH in the intestinal tissues collected from these patients. Unfortunately, the low number of cell profiles with productive infection identified in the examined tissue sections precluded drawing conclusions regarding the ability of panobinostat treatment to induce viral RNA production in the intestines.

### 2.7. Statistics

All statistical tests were performed using an alpha level of 0.05. Wilcoxon matched-pairs signed rank tests were utilized to assess whether there was a significant difference between the baseline and on-treatment measures.

## 3. Results and Discussion

### 3.1. Panobinostat Treatment Reduced Intestinal T Cell Activation* In Vivo* and Augmented IFN-*γ* Production by Intestinal T Cells Stimulated* Ex Vivo*

**Figure 2:**
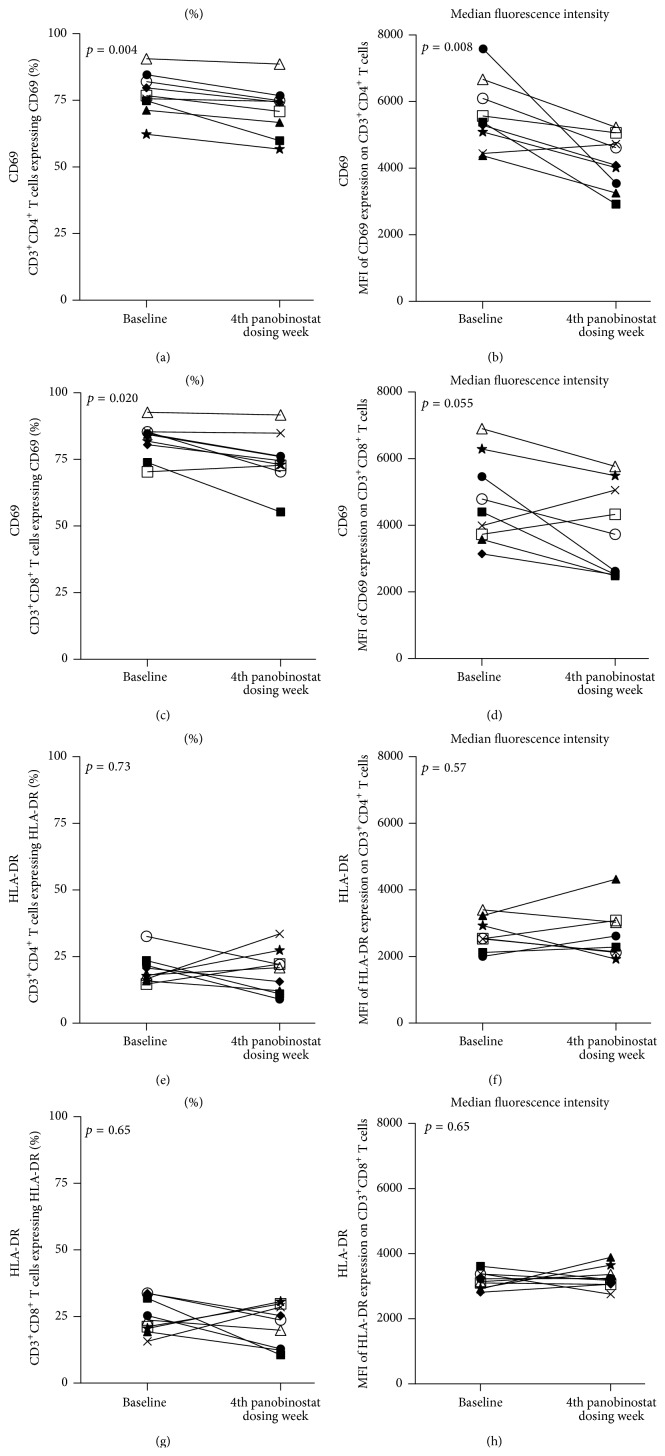
Panobinostat treatment was associated with reductions in the proportions of CD69^+^ intestinal T cells. (a–h) Flow cytometric analyses were performed on freshly isolated LPMCs. Intestinal CD4^+^ T cells (a-b; e-f) and CD8^+^ T cells (c-d; g-h) were assessed for CD69 (a–d) or HLA-DR (e–h) expression. The proportion of cells expressing the respective activation marker is graphed in the left column while the levels of marker expression are represented in the right column. Each patient participant is represented in all figures by the same distinct symbol. Wilcoxon matched-pairs signed rank tests were used to generate the reported *p* values.

Given our previous findings regarding the general anti-inflammatory activity of panobinostat in blood obtained from these patients [25, 31], we examined the activation status of freshly isolated LPMCs at baseline and during panobinostat treatment for surface expression of early (i.e., CD69) and late (i.e., HLA-DR) T cell activation molecules [32]. We observed a decrease in the proportion of CD69^+^ intestinal CD4^+^ and CD8^+^ T cells (*p* = 0.004, Figure 2(a); *p* = 0.020, Figure 2(c), resp.). Although we were unable to definitely determine whether this change resulted from decreased activation of resident cells versus an influx of naive cells, we did observe a significant decrease in the expression levels of CD69 on the intestinal CD4^+^ T cells (*p* = 0.008, Figure 2(b)) and a trend towards reduced CD69 expression on intestinal CD8^+^ T cells (*p* = 0.055, Figure 2(d)). In contrast to our findings regarding CD69 expression on intestinal T cells, we did not observe changes with panobinostat dosing in the proportions of HLA-DR expressing intestinal T cells or in the expression levels of HLA-DR on intestinal T cells (Figures 2(e) and 2(f)). It should be noted that these results from intestinal cells differ from PB outcomes from these patients [31]. Notable distinctions are that the baseline proportion of CD69^+^ T cells in the intestines is considerably higher than in the PB, as expected [33], and that the greatest increase in the proportion of activated CD4^+^ T cells was observed in PB during the first dosing week, while the observations made in the intestines are from the last dosing week. Thus, the differences between the observations in the intestines and PB regarding T cell activation may reflect differential stimulatory environments between these two anatomical compartments and the differential in the timing of the observations relative to the course of panobinostat treatment.

**Figure 3:**
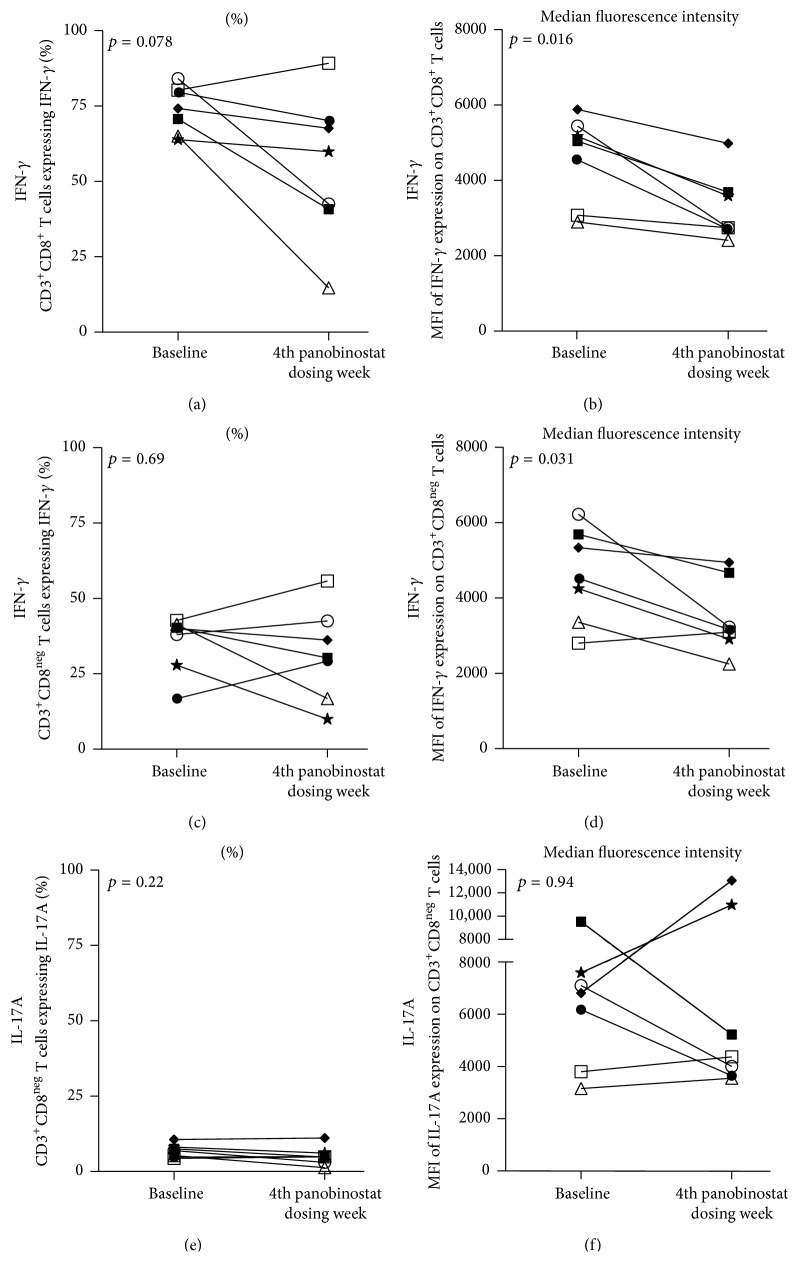
Intestinal T cells exhibit reduced intracellular IFN-*γ* expression following* ex vivo* stimulation. (a–f) LPMCs were stimulated* ex vivo* with PMA/ionomycin and then assessed for intracellular cytokine expression. Intestinal CD8^+^ T cells (a and b) and CD8^neg^ T cells (c–f) were assessed for INF-*γ* (a–d) or IL-17A (e and f) expression. The proportion of cells expressing the respective cytokine is graphed in the left column while the levels of cytokine expression are represented in the right column. Patient × and Patient ▲ were excluded from the intracellular cytokine expression analyses due low cell yields that precluded performance of the* ex vivo* stimulation either at the “baseline” or “during panobinostat” time point, respectively. Each patient participant is represented in all figures by the same distinct symbol. Wilcoxon matched-pairs signed rank tests were used to generate the reported *p* values.

Next, we performed an* ex vivo* mitogenic stimulation of LPMCs that were rested overnight following isolation. We examined CD8^+^ and CD8^neg^ T cells for intracellular expression of two proinflammatory cytokines (i.e., IFN-*γ* and IL-17A) [34]. We did not observe changes in the proportions of IFN-*γ*-producing intestinal T cells (Figures 3(a) and 3(c)). However, we did observe decreases in the expression levels of IFN-*γ* in both intestinal CD8^+^ and CD8^neg^ T cells (*p* = 0.016, Figure 3(b); *p* = 0.031, Figure 3(d), resp.). Overexpression of IFN-*γ* and other type 1 interferons in T lymphocytes has been observed both in experimental animal models of colitis and in patients with ulcerative colitis [35]. Thus, the detected reduction in IFN-*γ* expression could reflect a general pleiotropic anti-inflammatory effect of panobinostat on lamina propria-resident T lymphocytes. This reduction in expression does not readily conform to our observation that panobinostat treatment generally did not affect IFN-*γ* secretion by PB CD8^+^ T cell memory subsets from this patient cohort [36]. However, direct comparisons between these PB and intestinal cell analyses are challenging. The main reasons are the difference in timing of sample selection as noted above and the fact that the* ex vivo* stimulations were performed with staphylococcal enterotoxin B (SEB) antigen for the PB cells and PMA/ionomycin for the intestinal cells. Further, the blood analyses were stratified by memory subset providing different assay resolution. IL-17A production was not examined in the PB cells, but it was characterized for the intestinal CD8^neg^ T cells in response to mitogen stimulation. In this analysis, we did not observe cohort-wide changes in the proportion of cells expressing IL-17A or in the per cell expression (*p* = 0.22, Figure 3(e); *p* = 0.94, Figure 3(f), resp.). In accordance with the anticipated anti-inflammatory activity of panobinostat, these analyses demonstrate that panobinostat dosing generally reduced expression of the activation marker CD69 on intestinal T cells and reduced the capacity of intestinal T cells to produce IFN-*γ* in response to* ex vivo* stimulation.

### 3.2. The Number of IL-17A mRNA Producing Intestinal Cell Profiles Increased in the Epithelial Region with Panobinostat Dosing

**Figure 4:**
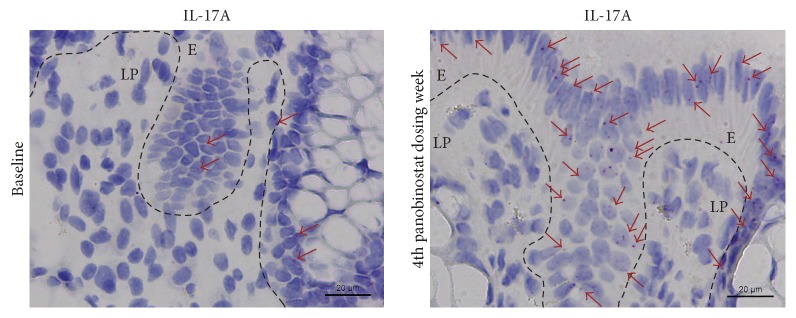
Intestinal IL-17A mRNA expression increased with panobinostat treatment. Representative images for IL-17A RNAScope ISH from Patient ★ are presented. Red arrowheads indicate a sampling of mRNA positive cell profiles in each image. Dashed lines demarcate lamina propria (LP) and epithelial (E) regions.

**Figure 5:**
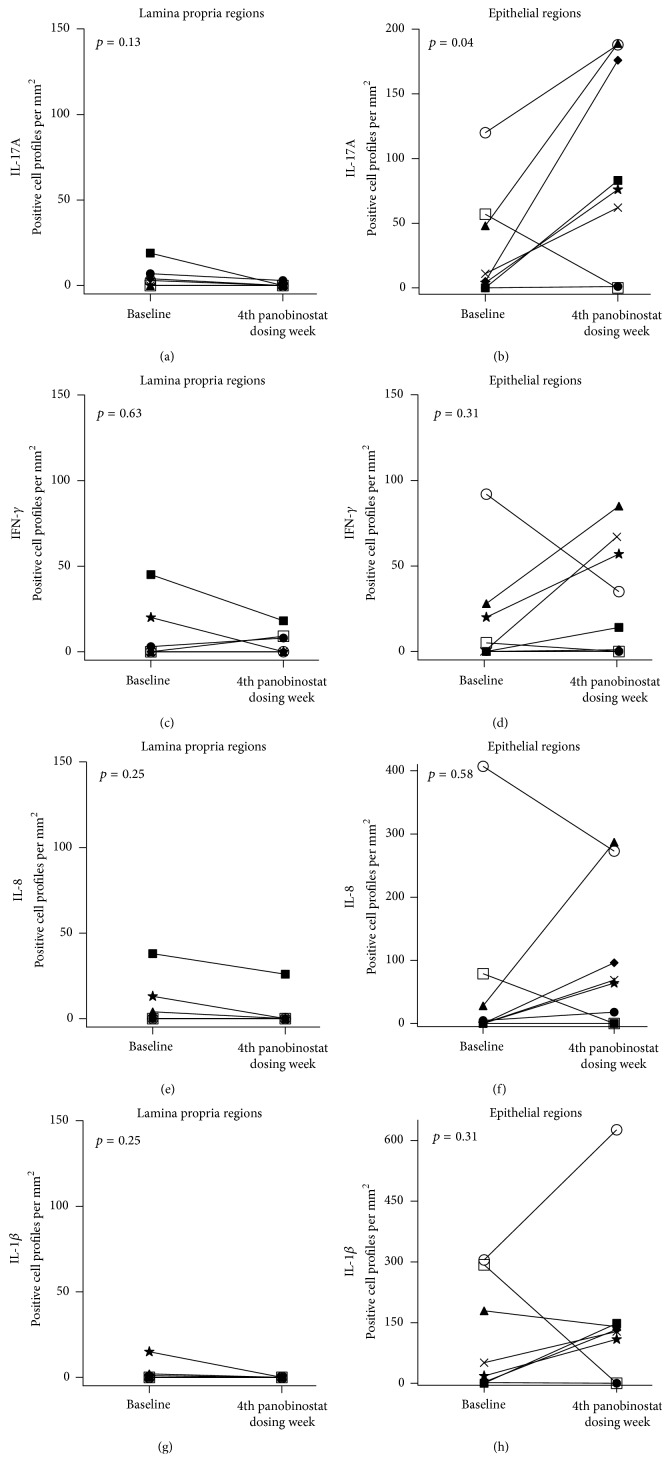
Epithelial region of intestines harbored the cells exhibiting IL-17A mRNA expression increase during panobinostat treatment.* In situ* hybridization for cytokine mRNA was performed with RNAScope technology. (a–h) Plots depict the number of cell profiles producing mRNA for IL-17A (a and b), IFN-*γ* (c and d), IL-8 (e and f), and IL-1*β* (g and h) at baseline and during panobinostat. Left column depicts positive cell profiles per mm^2^ within the lamina propria regions. Right column depicts positive cell profiles per mm^2^ within the epithelial regions. Patient ∆ was excluded from the final analyses due to insufficient tissue for both optimization and assay performance. Each patient participant is represented in all figures by the same distinct symbol. Wilcoxon matched-pairs signed rank tests were used to generate the reported *p* values.

To gain insights into changes in the numbers of individual cells producing cytokine mRNA in the intestine during panobinostat therapy, we performed quantitative RNA* in situ* hybridization using limited amounts of biopsy material with the assumptions that these tissue samples were representative for the whole intestine and that two-dimensional profile counts correlate with total cell counts. Analyses were stratified according to the anatomical region harboring the RNA expressing cells (i.e., lamina propria and epithelial regions). We found that panobinostat treatment was associated with a significant increase in the number of cell profiles (*p* = 0.04) exhibiting IL-17A production within the epithelial region but not the lamina propria (*p* = 0.13) (Figures 4, 5(a), and 5(b)). In contrast, we did not observe cohort-wide differences in the number of cell profiles exhibiting INF-*γ*, IL-8, or IL1*β* production in either the lamina propria or the epithelial regions of the intestine (Figures 5(c)–5(h)). The results for intracellular and* in situ* IL-17A expression (Figures 3(e), 3(f), 4, 5(a), and 5(b)) may appear to be contradictory at first glance. The likely explanation is that these observations highlight key methodological differences in the two assays. Specifically, the intracellular cytokine secretion assay was performed on isolated LPMCs. The sample preparation used for this analysis excluded the intraepithelial layer from assessment. In contrast, the* in situ* hybridization technique allowed quantitation throughout both the lamina propria and epithelial regions. Using this method and taking the stated assumptions into account, we were able to determine that the epithelial regions were the anatomical location of the increased IL-17A expression (Figure 5(b)).

IL-17A expression in the intestines is frequently associated with inflammatory processes, such as in inflammatory bowel disease [15, 37–41]. However, IL-17A also induces robust production of antimicrobial peptides important for maintaining the intestinal epithelial barrier [42]. IL-17A is produced by intestinal innate lymphoid cells to orchestrate mucosal barrier function [13], exhibits anti-inflammatory behavior in the context of cultured human colonic epithelial cells [43], and ameliorates inflammation in rodent colitis models [43, 44]. Thus the data presented here lead us to hypothesize that the panobinostat-induced upregulation in IL-17A mRNA observed in the intestinal epithelium is associated with mucosal barrier restoration. Such activity would be consistent with the general anti-inflammatory activity of panobinostat in these same patients [25], reduced LPMC T cell activation (Figures 2(a)–2(d)), reduced levels of IFN-*γ* production following* ex vivo* stimulation (Figures 3(a)–3(d)), and the unchanged intestinal IFN-*γ*, IL-8, and IL1*β* mRNA levels (Figures 5(c)–5(h)). Future studies are necessary to confirm that the panobinostat-induced IL-17A expression in the intestinal epithelium is indicative of mucosal barrier restoration and a reduction of chronic immune activation in HIV patients during suppressive ART. With such confirmation, perhaps panobinostat (or similarly acting compounds) could become therapeutic options for reducing morbidity associated with chronic inflammation in these individuals.

### 3.3. Conclusions

It is essential that conclusions are cautiously drawn from these data as they are derived from a single-arm phase I/II clinical study with increased peripheral blood cell-associated unspliced-HIV-1 RNA during panobinostat treatment as the primary endpoint. Nevertheless, these data do lend themselves to a key conclusion. Panobinostat has a clear biological impact in the intestines of HIV-1 patients as shown by the decreased CD69^+^ intestinal T cell frequency and increased IL-17A expression in the intestinal epithelium associated with panobinostat treatment. The significance of this last finding could be profound if future studies confirm that panobinostat dosing leads to improved mucosal barrier function in the intestines of HIV patients during suppressive ART.
